# Genetic Causes and Modifiers of Autism Spectrum Disorder

**DOI:** 10.3389/fncel.2019.00385

**Published:** 2019-08-20

**Authors:** Lauren Rylaarsdam, Alicia Guemez-Gamboa

**Affiliations:** Department of Physiology, Feinberg School of Medicine, Northwestern University, Chicago, IL, United States

**Keywords:** autism spectrum disorder, genetic modifiers, CNV, epigenetics, gene-environment interaction

## Abstract

Autism Spectrum Disorder (ASD) is one of the most prevalent neurodevelopmental disorders, affecting an estimated 1 in 59 children. ASD is highly genetically heterogeneous and may be caused by both inheritable and *de novo* gene variations. In the past decade, hundreds of genes have been identified that contribute to the serious deficits in communication, social cognition, and behavior that patients often experience. However, these only account for 10–20% of ASD cases, and patients with similar pathogenic variants may be diagnosed on very different levels of the spectrum. In this review, we will describe the genetic landscape of ASD and discuss how genetic modifiers such as copy number variation, single nucleotide polymorphisms, and epigenetic alterations likely play a key role in modulating the phenotypic spectrum of ASD patients. We also consider how genetic modifiers can alter convergent signaling pathways and lead to impaired neural circuitry formation. Lastly, we review sex-linked modifiers and clinical implications. Further understanding of these mechanisms is crucial for both comprehending ASD and for developing novel therapies.

## Introduction

Autism was first described by Kanner (1943) in a detailed report of 11 children with similar unusual tendencies. Intriguing common symptoms such as improper facilitation of language, indifference to other people, and obsessive interests can clearly be discerned while reading Kanner’s thorough patient history. Twenty-three years later, the first epidemiological study of autism estimated prevalence to be 4.5 per 10,000 individuals. Estimates have since increased drastically to 1 in 59 individuals affected, with at least three times as many males diagnosed as females (Loomes et al., 2017). This significant increase in prevalence is partially attributable to both increase in awareness and evolvement of Diagnostic and Statistical Manual of Mental Disorders (DSM) criteria, from a childhood form of schizophrenia in 1952, to a core diagnosis covering a spectrum of disorders in the present (Zeldovich, 2018). The changing landscape of factors required for diagnosis makes it difficult to quantify the actual increase in prevalence.

According to the current DSM-5 criteria, only two core features make up an autism spectrum disorder (ASD) diagnosis: (1) persistent deficits in social communication and social interaction across multiple contexts; and (2) restricted, repetitive patterns of behavior, interests, or activities (Lai et al., 2014). Because of the broad nature of these definitions, an ASD diagnosis often co-occurs with other conditions. Motor abnormalities (79%), gastrointestinal problems (up to 70%), epilepsy (up to 30%), intellectual disability (45%), and sleep disorders (50–80%) are common examples (Lai et al., 2014). Language disorders are frequently co-occurring and were even included in the DSM-IV criteria.

Since autism’s identification as a diagnosis, the medical and scientific community have put immense effort into determining the risk factors and etiology. In Kanner’s original assessment, he makes the unfortunate observation that in addition to patients having highly intelligent parents, “One other fact stands out prominently. In the whole group, there are very few really warm-hearted fathers and mothers” (Kanner, 1943). Thankfully, this “Refrigerator Mother” theory of autism was quickly disproved. ASD is now understood to be a disease of complex interaction between genetics and the environment, with heritability estimates ranging from 40 to 80% (Chaste and Leboyer, 2012). Extensive genetic studies have revealed hundreds of genes linked to autism. Epidemiological investigations have begun to elucidate which environmental factors might be contributing to risk, but there is a lot left to understand about how they interact with genetic predisposition to contribute to ASD etiology.

As is often the case with complex diseases, individuals with similar pathogenic variants may have drastically varying phenotypes. For example, people with duplications of proximal 15q range from unaffected to severely disabled (Cook et al., 1997; Bolton et al., 2001). Genetic modifiers – factors that modulate the expression of other genes – likely exist when individuals with the same pathogenic variant present on opposite ends of the spectrum. In this review, we will discuss what is presently known about the genetic landscape of ASD, then look at potential modifiers including copy number variation (CNV), double-hit mutations, epigenetic influences, and sex-linked effects.

## Genetics of ASD

### Identification of Candidate ASD Risk Genes

Following the classification of autism by Kanner, research efforts were undertaken to determine the disease etiology. Though it was initially assumed to be of environmental origin, an improved understanding of the role of genetics in human health soon suggested otherwise. In 1977, Folstein and Rutter (1977) conducted twin studies upon the observation that incidence among siblings was 50× higher than average. They found that monozygotic twins were more likely to share a diagnosis than dizygotic twins, suggesting a genetic influence. Bailey et al. (1995) supported this finding, documenting 60% concordance for monozygotic twins versus no concordant dizygotic pairs. In addition, risk of a child having ASD was found to be proportional to the percentage of the genome they shared with an affected sibling or parent (Constantino et al., 2010; Risch et al., 2014; Sandin et al., 2014). By the turn of the century, ASD was established to have some genetic component, though which genes were involved remained a mystery.

Early karyotype studies documenting chromosomal abnormalities began to shed light on which regions of the genome were involved (Gillberg and Wahlström, 1985). Additional susceptibility loci screens implicated regions on chromosome 7q, 1p, 3q, 16p, and 15q (IMGSAC, 1998; Barrett et al., 1999; Buxbaum et al., 2001; International Molecular Genetic Study of Autism Consortium [IMGSAC], 2001; Liu et al., 2001; Auranen et al., 2002; Lamb et al., 2002; Shao et al., 2003; Risch et al., 2014). However, to investigate at gene-level resolution, early studies had to use the candidate approach. Hypothesized targets included genes from suspected chromosomal regions that played a critical role in neurodevelopment, such as homeobox (*Hox*) family or *Wnt* genes. Unsurprisingly, many early studies using this method were largely inconclusive (Krebs et al., 2002; Lamb et al., 2002; Talebizadeh et al., 2002; Zhang et al., 2002). Starting in 2001, the candidate approach experienced moderate success with findings supporting reelin (*RELN*), aristaless related homeobox (*Arx*), methyl-CpG binding protein 2 (*MeCP2*), neuroligin 3 (*NLGN3*), neuroligin 4 (*NLGN4*), tuberous sclerosis complex 2 (*TSC2*), and ubiquitin protein ligase E3A (*UBE3A*)’s involvement in ASD etiology (Persico et al., 2001; Strømme et al., 2002; Carney et al., 2003; Jamain et al., 2003; Serajee et al., 2003; Jiang et al., 2004).

In the early 2000s, the advent of high throughput sequencing revolutionized genetic research and enabled investigators to study ASD on a genome-wide level. Sequencing technology quickly confirmed that the etiology of ASD was multigenic and highly heterogeneous, with very few of the same pathogenic variants present in a significant percentage of afflicted individuals. It is now known that the average case is a product of many susceptibility-increasing variations. Only a handful of ASD-related diseases have monogenic causes, such as Rett syndrome, fragile X syndrome, tuberous sclerosis, and Schuurs–Hoeijmakers syndrome (Artuso et al., 2011; Stern et al., 2017; Woodbury-Smith and Scherer, 2018). Dozens of large-scale genetic studies have since been conducted on ASD patients and their families, leading to hundreds of risk genes being identified. While these proteins have diverse functions, a majority of reproducible hits come from two broad classes of proteins: those involved in synapse formation, and those involved in transcriptional regulation and chromatin-remodeling pathways (De Rubeis et al., 2014).

Synapse-related risk genes include those encoding cell-adhesion proteins such as neuroligins, neurexins, and cadherins; synaptic vesicle cycling proteins synapsin-1 (SYN1) and synapsin-2 (SYN2); ion transport proteins such as sodium voltage-gated channel alpha subunit 2 (SCN2A), calcium voltage-gated channel subunit alpha1 E (CACNA1E), calcium voltage-gated channel auxiliary subunit beta 2 (CACNB2), potassium voltage-gated channel subfamily Q members 3 and 5 (KCNQ3 and KCNQ5), potassium voltage-gated channel subfamily D member 2 (KCND2), glutamate receptor signaling protein SH3 and multiple ankyrin repeat domains 3 (SHANK3), synaptic Ras GTPase activating protein 1 (SYNGAP1), and gamma-aminobutyric acid type A receptor gamma3 subunit (GABRG3) (Jamain et al., 2003; Durand et al., 2012; Schmunk and Gargus, 2013; Giovedí et al., 2014; Stessman et al., 2017). *In vivo* data supports the implication of synapse pathology and abnormal neural network formation in ASD.

Additional susceptibility loci impact transcription of other proteins through various mechanisms. For example, multiple studies have found an increased *de novo* mutation load in regulatory elements of ASD risk genes in patients (Turner et al., 2016, 2017; Short et al., 2018). The broad class of susceptibility genes that impacts transcription and chromatin-remodeling pathways includes *MeCP2, UBE3A*, chromodomain helicase DNA binding protein 8 (*CHD8*), activity dependent neuroprotector homeobox (*ADNP*), pogo transposable element derived with ZNF domain (*POGZ)*, fragile X mental retardation protein (*FMRP*), and RNA binding forkhead box (*RBFOX*) genes (Carney et al., 2003, p. 2; Samaco et al., 2005; De Rubeis et al., 2014; Stessman et al., 2017; Tran et al., 2019). These pathogenic variants have the potential to induce extremely widespread effects. For example, Tran et al. (2019) recently showed that *FMRP* and fragile X related protein 1 (*FXRP1*) mutations can result in abnormal RNA-editing enzyme activity, resulting in a global bias for adenosine-to-inosine hypoediting in ASD brains. Diverse phenotypes that may result are further discussed in the epigenetics section.

#### Somatic Mosaicism and ASD Risk

Disease-causing variations were conventionally thought to be familial/inherited and present in every cell in the body. However, the role of somatic mosaicism, which is the result of a post-zygotic DNA mutation, is increasingly being recognized as crucial to various neurodevelopmental diseases including autism (Poduri et al., 2013; Ronemus et al., 2014; D’Gama and Walsh, 2018). During neurogenesis, each progenitor gives rise to roughly five single nucleotide variants (SNV) per day as the brain rapidly develops (Bae et al., 2018; D’Gama and Walsh, 2018). Studies estimate that of *de novo* pathogenic variations, roughly 5–7% are postzygotic, though estimates of up to 22% have been reported (Acuna-Hidalgo et al., 2015; Freed and Pevsner, 2016; Krupp et al., 2017; Lim et al., 2017). Most mutations are harmless, but variations in exons can be extremely detrimental. Pathogenic somatic variations have been connected to ASD, Rett syndrome, tuberous sclerosis, intellectual disability, schizophrenia, and many other disorders (Clayton-Smith et al., 2000; Bourdon et al., 2001; Qin et al., 2010; Gilissen et al., 2014; Acuna-Hidalgo et al., 2015; Tyburczy et al., 2015; Freed and Pevsner, 2016; Dou et al., 2017; Doyle et al., 2017; Krupp et al., 2017; D’Gama and Walsh, 2018).

Until recently, our understanding of somatic mosaicism in ASD was restricted primarily to case reports (Oliveira et al., 2003; Sauter et al., 2003; Papanikolaou et al., 2006; Havlovicova et al., 2007; Yurov et al., 2007; Castermans et al., 2008; Kakinuma et al., 2008; Vorstman et al., 2011). Several recent investigations of whole exome-sequencing (WES) data from large cohorts have been instrumental in shaping our understanding of the role of somatic mosaicism, which is currently estimated to account for roughly 3–5% of simplex ASD cases (Freed and Pevsner, 2016; Krupp et al., 2017). Lim et al. (2017) used WES analysis of 5,947 ASD-affected families and determined that somatic variations in autistic individuals were more likely to be in critical exons than variations in control siblings. Interestingly, they found that the pathogenic variants had enhanced expression in the amygdala, an area critical for emotional response and social awareness (Rasia-Filho et al., 2000). In another large WES study, new risk genes identified were enriched in the cerebellum, which suggests potential coordination difficulty that could be related to gait disorders common in autistic children (Dou et al., 2017). Freed and Pevsner (2016) analyzed 2,388 families and identified an ascertainment bias for pathogenic mosaic variations in ASD individuals relative to unaffected siblings. These large-scale sequencing studies of post-zygotic mutations have both confirmed previously implicated candidate genes, such as *SCN2A*, in addition to revealing dozens of new risk genes and establishing somatic mosaicism as a significant factor in ASD etiology (Lim et al., 2017).

#### CNVs Contribute to ASD Susceptibility

Copy number variations (CNVs) are submicroscopic structural variants in chromosomes that include duplications, deletions, translocations, and inversions, sometimes stretching several kilobases (Marshall et al., 2008). CNV can either be inherited or arise *de novo* (Thapar and Cooper, 2013). Many genes may be affected with these changes, but not all are necessarily drivers of disease. Studies have found a higher load of rare, genic CNVs in autistic individuals, implicating these variants in ASD pathology (Sebat et al., 2007; Pinto et al., 2010; Pizzo et al., 2019). CNV is now understood as an extremely important contributing factor in ASD susceptibility, and current estimates postulate that these variations directly cause roughly 10% of ASD cases (Geschwind, 2011).

Studies of how individual CNVs contribute to ASD have been done for more frequent structural variants, such as 16p11.2 duplications. The majority of the 25 genes in this region are highly active during nervous system development and are critical for proper formation (Blaker-Lee et al., 2012). While the alteration of many genes involved in development suggests a mechanism for the diverse symptoms observed in ASD, Golzio et al. (2012) reported that only one gene in the 16p11.2 region, potassium channel tetramerization domain containing 13 (*KCTD13*), seems to be the major driver for neuropsychiatric disease. Duplications or deletions of this gene are thought to affect synaptic transmission through altered regulation of Ras homolog family member A (*RHOA*) (Escamilla et al., 2017). However, Escamilla et al. (2017) also hypothesized that *KCTD13* deletions alone are not likely to be sufficient for disease. Mouse models suggest another gene in the 16p11.2 region as a driver of disease – mitogen-activated protein kinase 3 (*MAP3*) – with deletions resulting in altered cortical cytoarchitecture and reduced brain size (Pucilowska et al., 2015). Likely, the real driver of disease in 16p11.2 duplications or deletions is not from just one gene, but an interaction of all 25 contributing to susceptibility. Iyer et al. (2018) systematically investigated interaction between genes in the 16p11.2 region, using RNAi in *Drosophila* to test 565 pairwise knockdowns. In addition to 24 modifying interactions discovered between pairs of genes within the 16p11.2 region, they also found 46 interactions between 16p11.2 genes and others involved in neurodevelopment (Iyer et al., 2018). This strongly suggests that modifying interactions within CNVs result in the complex phenotypes observed and may not be elucidated from studies with single genes, a phenomenon that is likely true for other CNV regions in addition to 16p11.2.

The disease mechanisms of other CNVs are less frequently studied due to the paucity of commonly affected regions. Even the most prevalent ASD-associated CNVs, such as 15q11-13 as well as 16p11.2, are only present in roughly 1% of autism cases (Kumar et al., 2008; Marshall et al., 2008; Weiss et al., 2008; Marshall and Scherer, 2012). In addition, there are no known CNVs with complete penetrance; studies that find CNVs with significant correlation to ASD often detect non-ASD carriers, or ASD siblings without the variant (Marshall et al., 2008). One useful approach in the midst of this heterogeneity is to assess common functional networks affected. Repeatedly, studies have shown that autistic individuals have deletions in synaptic genes, such as *SHANK3*, dipeptidyl peptidase-like 10 (*DPP10*), neuroligins, and neurexins (The Autism Genome Project Consortium et al., 2007; Marshall et al., 2008; Glessner et al., 2009; Pinto et al., 2010, 2014; Marshall and Scherer, 2012). Other common functional gene sets with rare CNVs include those involved in cell proliferation and development, chromatin regulation, and ubiquitin pathways (Glessner et al., 2009; Pinto et al., 2010, 2014).

With certain CNVs, copy number dosage appears to affect disease phenotype. For example, Horev et al. (2011) observed a dose-dependent effect and change in brain structure in mice with 16p11.2 deletions and duplications, but this effect is not as established in humans (Kumar et al., 2008). Another study investigating CNV in the locus containing the *UBE3A* gene also report a positive correlation between duplication and autistic traits in mice, as well as decreased glutamatergic synaptic transmission (Smith et al., 2011). In humans, Stefansson et al. (2014) analyzed a 15q11.2 CNV region of autistic individuals and found two brain areas with dose-dependent structural and functional effects. Interestingly, some non-ASD/schizophrenic controls who were diagnosed with dyslexia and dystaxia also exhibited the same structural changes (Stefansson et al., 2014). In another study with humans, Girirajan et al. (2013) reported a dose-dependent effect from their microarray analysis with identified CNVs in ASD-associated genes, finding a positive correlation between duplication size increase and autism severity increase, but no correlation between duplication size and non-verbal IQ. CNV are often critical and complex contributors to ASD risk, but patients with similar structural variants may have highly variable phenotypes. Following sections will discuss how non-causative modifiers play an important role in modulating CNV pathogenicity.

#### Epigenetic Regulation and ASD

Genes with epigenetic-modulating functions are highly involved in ASD susceptibility. A recent review of 215 candidate genes estimated that 19.5% are epigenetic regulators, suggesting the potential for diverse disease phenotypes from few pathogenic variants (Duffney et al., 2018). Another study suggested that risk genes with high penetrance were typically located in the nucleus and involved in modulation of expression, or tied to the protein-protein interaction network essential in guiding CNS developmental patterning (Casanova et al., 2016). Twin studies particularly demonstrate the profound ways epigenetics can modulate disease phenotype; for example, a study of 50 pairs of monozygotic twins discordant for ASD reported numerous autism-associated differentially methylated regions, with methylation patterns at some CpG sites common to symptom groups (Wong et al., 2014).

Though the scientific and medical community still has a great deal to learn about epigenetic modulation of ASD, patterns have emerged from large-scale epigenomic studies. Susceptibility loci often include genes involved in methylation such as *KMT2C*, lysine methyltransferase 5B (*KMT5B*), and lysine demethylase 6B (*KDM6B*); chromatin remodeling proteins including MeCP2, CHD8, and POGZ; RNA-binding/splicing proteins such as FMRP and the RBFOX family, post-translational modification proteins like UBE3A, mindbomb E3 ubiquitin protein ligase 1 (MIB1); or transcription factors like ADNP and additional sex combs like 3 (ASXL3) (De Rubeis et al., 2014). Targets of these proteins can range from few to hundreds, and often include pathways previously implicated in autism, such as synaptic formation. To demonstrate how mutations in a single epigenetic regulator can modify many other risk genes, we will look more in depth at two key susceptibility genes: *MeCP2* and *UBE3A*.

MeCP2 is a chromatin modifier that is consistently implicated in ASD. In a healthy individual, the binding action of MeCP2 has been shown to regulate many genes with synaptic function, such as *GABRB3*, brain derived neurotrophic factor (*BDNF*), distal-less homeobox 5 (*DLX5*), insulin like growth factor binding protein 3 (*IGFBP3*), cyclin dependent kinase like 1 (*CDKL1*), protocadherin beta 1 (*PCDHB1*), protocadherin 7 (*PCDH7*), and lin-7 homolog A (LIN7A) (Samaco et al., 2005; Kubota and Mochizuki, 2016). It also serves post-translational functions (Cheng and Qiu, 2014). In addition, MeCP2 is the rate-limiting factor in regulating glutamatergic synapse formation during development, which implicates its involvement in yet another important aspect of ASD pathology (Chao et al., 2007). MeCP2 is shown to be reduced in the frontal cortex of ASD individuals due to increased methylation of its promoter (Samaco et al., 2005; Nagarajan et al., 2006, 2008).

UBE3A, an E3 ubiquitin protein ligase, is a second important epigenetic regulator strongly implicated in ASD pathology. It is modulated by MeCP2, but can be causative on its own (Samaco et al., 2005, p. 2). *UBE3A* lies in the chromosomal region 15q11-13, which is commonly duplicated in autism. Dose-dependent effects have been positively correlated with reduced excitatory synaptic transmission, delay of first word, and psychomotor regression (Guffanti et al., 2011; Smith et al., 2011; Xu et al., 2018). The mechanism of UBE3A’s pathological activity can be hypothesized based on its function as a ubiquitin ligase, which targets proteins for degradation, but research is still revealing exactly how these dose-dependent impairments occur. Lee et al. (2014) identified four proteosome-related proteins that were direct substrates of UBE3A. Overexpression of *UBE3A* and one of its substrates, proteasome 26S subunit, non-ATPase 4 (*Rpn10*), led to increased accumulation of ubiquitinated proteins, suggesting a proteostatic imbalance. Proteosome health has been strongly implicated in dendritic spine outgrowth, linking UBE3A with one of the key pathologies observed in autism (Hamilton et al., 2012; Puram et al., 2013). Its involvement in Wnt signaling could also cause significant perturbation during development (Yi et al., 2017). *MeCP2* and *UBE3A* are just two examples of how one altered gene can have extremely far-reaching effects.

Large-scale epigenetic studies have also helped achieve a broader picture of epigenetic mis-regulation in ASD. Sun et al. (2016) conducted a histone acetylome-wide association study on 257 post-mortem prefrontal and temporal cortex samples. Surprisingly, they found that >68% of both syndromic and idiopathic cases shared a common acetylome signature at roughly 5,000 enhancer regions (Sun et al., 2016). Intriguingly, a SHANK3 mouse model of autism displayed rescued behavioral phenotypes when treated with a potent histone deacetylase inhibitor, reinforcing the role of epigenetics in ASD (Qin et al., 2018). Ladd-Acosta and coworkers measured over 485,000 CpG loci in post-mortem brain tissue from 40 individuals and identified four differentially methylated regions. Three sites were found in cortical tissue: the proline rich transmembrane protein 1 (*PRRT1*) 3’ UTR, promoter regions of tetraspanin 32 (*TSPAN32*), and *C11orf21.* The last site, an alternative promoter for succinate dehydrogenase complex flavoprotein subunit A pseudogene 3 (*SDHAP3*), was found in cerebellar tissue (Ladd-Acosta et al., 2014). Affected pathways implied in these studies and others include synaptic transmission, immune function, ion transport, and GABAergic genes (Nardone and Elliott, 2016; Sun et al., 2016; Andrews et al., 2017; Zhubi et al., 2017).

Mor et al. (2015) took a different approach, using small RNA sequencing data and correlating results to genome-wide DNA methylation data to find dysregulated miRNAs. miRNAs that were found to be significantly expressed in the ASD brain were linked to synaptic function, consistent with data from numerous other studies. They also discovered a link to the oxytocin receptor (*OXTR*) gene, suggesting attenuated *OXTR* expression in the autistic brain. This finding was supported by a study that found fetal membranes from preterm birth had hypermethylated *OXTR*, potentially linking an environmental risk factor to a pathological mechanism (Behnia et al., 2015). Another risk gene with epigenetic functions is engrailed homeobox 2 (*EN2*), a homeobox gene with an unusual methylation pattern in ASD that has been hypothesized to cause abnormal cerebellar Purkinje growth (James et al., 2013). The list of ASD risk genes with epigenetic functions is vast, suggesting a mechanism by which few mutations can result in widespread misregulation of gene expression. Because of this, genes with epigenetic functions and their substrates may be promising targets of therapies. For example, mutations in *FMRP*, a chromatin remodeler, result in widespread gene expression abnormalities, but a recent study found that inhibition of FMRP target bromodomain containing 4 (*BRD4*) alleviated many of the disease characteristics (Korb et al., 2017). Proteins with epigenetic-regulating function may also be key targets of disease modifiers, a concept that will be discussed later in this review.

### ASD Risk Genes Overlap With Other Diseases

Large-scale sequencing studies of major psychiatric diseases have revealed extensive overlap in risk loci, challenging the classification of these conditions as distinctive disorders. In 2013, the Cross-Disorder Group of the Psychiatric Genomics Consortium (PGC) conducted a massive study with 33,332 cases and 27,888 controls in order to identify pathogenic variants shared between ASD, schizophrenia, bipolar disorder, ADHD, and major depressive disorder (Cross-Disorder Group of the Psychiatric Genomics Consortium, 2013; Cross-Disorder Group of the Psychiatric Genomics Consortium et al., 2013). In addition to establishing varying degrees of pair-wise crossover, they found loci that reached genome-wide significance for all five disorders near the following genes: inter-alpha-trypsin inhibitor heavy chain 3 (*ITIH3*), arsenite methyltransferase (*AS3MT*), calcium voltage-gated channel subunit alpha1 C (*CACNA1C*), and *CACNB2*. Glessner et al. (2017) have also conducted a large-scale meta-analysis of structural variants across the same diseases and correlated structural variants in the loci of dedicator of cytokinesis 8 (*DOCK8*) and KN motif and ankyrin repeat domains 1 (*KANK1*) with all five conditions. Schork et al. (2019) recently hypothesized that abnormal gene regulation in radial glia and interneurons during mid-gestation is a mechanism of shared risk, after using GWAS to identify susceptibility loci in genes including phosphodiesterase 1A (*PDE1A*), protein phosphatase 1 regulatory inhibitor subunit 1C (*PPP1R1C*), *RHOA*, immunoglobulin superfamily member 11 (*IGSF11*), and sortilin related VPS10 domain containing receptor 3 (SORC3).

Studies also report shared susceptibility genes across a more restricted set of psychiatric diseases. For example, ASD, intellectual disability (ID), and schizophrenia have been found to share risk loci in FMRP targets, *CHD5*, *CHD8*, *SCN2A*, and neurexin 1 (NRXN1) (Iossifov et al., 2014; Wang et al., 2019). Wang et al. (2019) also found commonalities across ASD, ID, and bipolar disorder with increased incidence of *de novo* pathogenic variants in periodic circadian regulator 1 (*PER1*) and lysine methyltransferase 2C (*KMT2C*). Khanzada et al. (2017) found 23 susceptibility genes common to ASD, bipolar disorder, and schizophrenia including dopamine receptor D2 (*DRD2*), cholinergic receptor nicotinic alpha 7 subunit (*CHRNA7*), 5-hydroxytryptamine receptor 2A (*HTR2A*), solute carrier family 6 member 3 (*SLC6A3*), and tryptophan hydroxylase 2 (*TPH2*). Hit genes were primarily involved in dopamine and serotonin homeostasis, suggesting a potential mechanism for abnormal emotional regulation observed across all three disorders (Khanzada et al., 2017). The immense crossover revealed in these studies intriguingly suggests some level of shared etiology across psychiatric conditions, despite having clinically distinct presentations.

Of the four other diseases assessed in the PGC study, the most highly correlated disease to ASD was schizophrenia (Cross-Disorder Group of the Psychiatric Genomics Consortium et al., 2013). Previous epidemiological studies had suggested their linkage, reporting increased risk of ASD in children with schizophrenic parents and significant co-morbidity of child-onset schizophrenia and autism (Rapoport et al., 2009; Sullivan et al., 2012). A follow-up report to the 2013 PGC study estimated genetic correlation between the two diseases to be 23%, with shared risk loci including several genes involved in neurodevelopment, such as forkhead box P1 (*FOXP1*), exostosin glycosyltransferase 1 (*EXT1*), astrotactin 2 (*ASTN2*), mono-ADP ribosylase 2 (*MACROD2*), and histone deacetylase 4 (*HDAC4*) (The Autism Spectrum Disorders Working Group of The Psychiatric Genomics Consortium, 2017). In addition to susceptibility genes involved in neurodevelopment, other studies have also reported shared susceptibility in genes affecting chromatin remodeling, oxidative stress response, and lipid metabolism (McCarthy et al., 2014; Lim et al., 2017).

Many studies have also found a significant correlation between autistic and ADHD scored traits. This includes a study of autistic symptoms in ADHD probands and siblings, autistic trait correlation in an ADHD twin sample, and an association between autistic and ADHD traits in the general population (Reiersen et al., 2007; Ronald et al., 2008; Mulligan et al., 2009; Stergiakouli et al., 2017). Nijmeijer et al. (2010) identified five specific genetic loci that were associated with ASD traits in children with ADHD: 7q36, 16p13, 18p11, 15q24, and 12q24. A study investigating the overlap of pathological structural variants in ADHD and ASD found significant overlap in genes related to a wide variety of processes, including the nicotinic receptor signaling pathway and cell division (Martin et al., 2014). The shared heritability of ASD and ADHD is still being explored, and is further discussed in a review by Rommelse et al. (2010).

Since ASD is a multigenic and highly heterogeneous disease that often co-occurs with other conditions, it can be difficult to distinguish which genes truly have overlapping risk for multiple psychiatric conditions, and which variations are responsible for the common disease phenotypes. For example, the ubiquitin ligase gene *UBE3A* is implicated in both autism and Angelman Syndrome, a condition distinct from ASD but with similar symptoms, such as movement and speech defects. Interestingly, Angelman Syndrome is generally associated with *UBE3A* deletions, while ASD can be caused by duplications – yet the same individual can be diagnosed with both syndromes (Peters et al., 2004; Williams et al., 2010; Smith et al., 2011; Kalsner and Chamberlain, 2015; Yi et al., 2015). Another example is intellectual disability, which co-occurs with autism in roughly 45% of cases (Lai et al., 2014). Multiple studies have found that ASD and intellectual disability share risk loci (Pinto et al., 2010; McCarthy et al., 2014), but overlapping phenotypes are a potentially confounding factor. Similarly, other risk genes for ASD are epigenetic regulators whose effectors are associated with different diseases (Samaco et al., 2005; Pinto et al., 2010; Michaelson et al., 2017). The interaction and overlap between psychiatric disorders is complex, and much is left to discern regarding shared disease mechanisms.

## Modifiers in ASD

### Genetic Modifiers

Though significant progress has been made in determining genetic causes of ASD, many aspects of how pathogenic variants regulate genetic susceptibility remain unknown. Individuals with the same variants can have widely heterogeneous disease presentations and levels of disability. Presence of second modulating variants that may interact with other susceptibility loci are one possible explanation of this heterogeneity. This “second hit” could be somatic – a phenomenon first proposed to cause disease by Alfred Knudson in the context of retinoblastomas – or in the germline, a “two-locus model” previously explored in conditions such as Hirschprung disease (Knudson, 1971; Fisher and Scambler, 1994; McCallion et al., 2003). To date, genetic evidence supporting a multiplex theory of autism has primarily been found for germline second-hits. Studies with CNVs will be discussed first, followed by a brief overview of known modulating SNPs. These investigations of how non-causative variants may modify the ASD phenotype are challenging to undertake, as few autistic individuals have the same pathogenic variants. In addition, there is not yet a complete understanding of which CNVs and SNPs are pathogenic in ASD.

One way to circumvent these issues is to investigate an autism subtype with a monogenic cause, such as Rett Syndrome. Artuso et al. (2011) used this strategy and identified 15 “likely” and 14 “unlikely” modulators of the RTT phenotype based on array comparative genome hybridization with eight RTT subjects. Another valuable approach is to assess monozygotic twins with a discordant phenotype. Several studies have assessed potential differences in CNVs or epigenetic regulation in discordant monozygotic twins, revealing potential methylation pattern differences in one case and anomalies in the 2p25.3 region in another (Bruder et al., 2008; Kunio et al., 2013; Rio et al., 2013). However, a study involving 100 twin pairs failed to find differences in CNVs that could explain the discordant phenotypes (Stamouli et al., 2018). The authors still acknowledge postzygotic mosaicism as a potential modifier and encourage more studies to help develop a clearer understanding of CNV modulating activity.

A handful of reports also exist of putative modifying CNVs in polygenic ASD cases with unrelated subjects. For example, Girirajan et al. (2012) found that children with two CNVs not known to be pathological were eight times more likely to be diagnosed with developmental delay than controls. In the same year, a study of *SHANK2* pathogenic variants found abnormalities in both individuals with neuropsychiatric disease and controls, suggesting the presence of additional variants in order to cause disease. Three of the patients with *de novo SHANK2* mutations were also found to have deletions of *CHRNA7* and cytoplasmic FMR1 interacting protein 1 (*CYFIP1*) – both previously implicated in ASD – supporting a “multiple-hit” model of autism (Leblond et al., 2012). *CHRNA7* was also suggested as a potential modifier in an earlier study by Szafranski et al. (2010). Barber et al. (2013) provided further support for a multiple-loci model of ASD upon finding that patients with 16p12.1 duplications had a more severe phenotype when a second large CNV was present. Included in these hypothesized modifier regions were genes G protein regulated inducer of neurite outgrowth 2 (*GPRIN2*) – previously implicated as a modifier in the study by Artuso et al. (2011) – and steroid sulfatase (*STS*), which was formerly thought to be non-causative (Li et al., 2010). More recently, an analysis of 20,226 patient records revealed 19 patients with CNVs in contactin 6 (*CNTN6*), a gene hypothesized to be involved in neurodevelopmental disorders including ASD (Repnikova et al., 2019). The authors were not able to find any significant genotype-phenotype relationships and concluded that CNV in *CNTN6* were likely benign or modifying, but not causative of disease.

In addition to CNVs, there may be thousands of smaller pathogenic variants – such as SNPs and indels – that also modulate severity. For example, in a study of developmental delay, individuals that only carried a specific 16p12.1 microdeletion had a less severe phenotype than individuals with random second variants (Girirajan et al., 2010). One study of individuals with 22q11.2 deletion syndrome – all haploinsufficient for an mGluR network gene – found that 20% who were co-diagnosed with autism had second-hit pathogenic variants, while only 2% of 22q11DS individuals without autism had second hits (Wenger et al., 2016). Bonnet-Brilhault et al. (2016) assessed a family affected with ID and ASD due to *NLGN4X* pathogenic variants and found that individuals with ASD – but not ID or controls – had second-hit variants in glycine receptor beta (*GRLB*) and ankyrin 3 (*ANK3*). Additional evidence may exist, but GWAS and WES studies have tended to focus on causative susceptibility loci. Therefore, other variants which are not causative by themselves are not often emphasized or even reported. The emerging study of all types of genetic modifiers is a relatively recent development, and continuing advancements in sequencing technology, analyzing software, and expansion of databases should lay the framework for significant advancements in the near future.

### Epigenetics and the Environment

Autism susceptibility is currently estimated to be 40–80% genetic. Environmental factors – likely acting through epigenetic regulation as the major mechanism – presumably compromise the remainder of the risk. Hundreds of potential environmental factors have been suggested to contribute to risk, such as increased parental age (especially paternal), maternal complications or infections during pregnancy, or prenatal exposure to anticonvulsants (Rasalam et al., 2005; Kong et al., 2012; O’Roak et al., 2012; Ohkawara et al., 2015). In-depth reviews of these findings can be found elsewhere (Gardener et al., 2009; Chaste and Leboyer, 2012; Liu et al., 2016; Karimi et al., 2017; Modabbernia et al., 2017; Bölte et al., 2019). In this review, we will only discuss the epigenetic modifying effects of valproic acid – an anticonvulsant – as one example of the widespread modifications that an environmental factor can induce. Valproic acid has been hypothesized to modify gene expression through histone deacetylase inhibition activity and is sometimes used to induce an autistic phenotype in animal models (Kataoka et al., 2013). Examples of its far-reaching effects include apoptotic cell death in the neocortex, decreased proliferation in the ganglionic eminence, increased homeobox A1 (*HOXA1*) expression, abnormal serotonergic differentiation via Achaete-Scute family BHLH transcription factor 1 (*ASCL1*) silencing, disrupted serotonin homeostasis in the amygdala, dendritic spine loss, reduced prefrontal dopaminergic activity, and disruption of the glutamatergic/GABAergic balance (Stodgell et al., 2006; Dufour-Rainfray et al., 2010; Kataoka et al., 2013; Wang et al., 2013; Jacob et al., 2014; Takuma et al., 2014; Hara et al., 2015; Iijima et al., 2016; Mahmood et al., 2018).

In more thorough studies of the mechanism of action, Go et al. (2012) found that rats exposed to valproic acid *in utero* presented enhanced proliferation of neural progenitors and delayed neurogenesis by upregulating *Wnt1* expression and activating the GSK-3β/β-catenin pathway, leading to macrocephaly. Another study found that valproic acid increased BDNF by two transcriptional mechanisms involving *MeCP2* and tissue plasminogen activator (tPA). This increase in BDNF is proposed to alter neurite outgrowth, impairing synapse formation (Ko et al., 2018). Finally, Kolozsi et al. (2009) observed a downregulation of *NLGN3* – a highly implicated autism risk gene involved in synapse formation – in both hippocampal and somatosensory cortex of valproate-exposed mice. Examples of other proposed environmentally modulated mechanisms of ASD risk exist, but the literature supporting valproic acid is an excellent example of the heterogeneous effects one environmental factor can induce. Further research is strongly needed to determine how the environment modulates ASD risk.

Clearly, epigenetics can have a profound impact on the transcriptome of an organism. Pathogenic variants in even one epigenetic-regulating gene or effects from the environment can cause widespread gene dysregulation. Epigenetic modulators can themselves be causative of disease, but they may also exacerbate or ameliorate the disease phenotype by influencing expression of risk genes. More genome-wide studies are needed to understand the common ASD epigenome, and whether certain epigenetic markings might be protective or detrimental to individuals who are genetically susceptible. In addition, more studies are needed to decipher epigenetics as a link between environmental risk factors and genetic susceptibility. There is a possibility that certain environmental factors could have protective epigenetic effects, providing potential avenues for therapy.

### Sex-Linked Modifiers

It is well established that ASD affects males at much higher rates than females. The reasons for this are not yet completely clear. Some studies argue that differential expression between genders may result in an under-diagnosis of females, as males tend to present more external behavior (e.g., aggression or increased repetitive behavior) and females tend to present more internal behavior (e.g., depression and avoiding demands) (Werling and Geschwind, 2013). While this may contribute to the rates of diagnosis, other possibilities include that the female sex is protective and/or males are particularly vulnerable. This may be due to influence from hormones, genetics, or other unknown factors. The genetically heterogeneous nature of ASD makes it likely that all these elements are involved – sex bias varies drastically based on factors such as which CNVs are causative or which comorbidities are present, suggesting diverse means by which a sex bias may occur (Amiet et al., 2008; Polyak et al., 2015). Potential mechanisms of sex-specific modulation will be discussed briefly, although more thorough reviews are available elsewhere (Ferri et al., 2018).

Multiple studies argue that the female sex is protective toward ASD susceptibility (Robinson et al., 2013; Pinto et al., 2014). For example, the average mutational burden in diagnosed females is much higher than in males, suggesting that males have a lower mutational burden threshold (Jacquemont et al., 2014; Desachy et al., 2015). Another study by Robinson et al. (2013) investigated nearly 10,000 dizygotic autistic twin pairs and found that siblings of female probands had significantly worse symptoms than siblings of male probands. Many investigations have also found that unaffected mothers may carry the same mutation as their affected male children. One particularly well-documented example for this is the 15q11-13 duplication (Cook et al., 1997; Schroer et al., 1998; Gurrieri et al., 1999; Boyar et al., 2001). This region codes for GABA_*A*_ receptors, which is supported by the observation of perturbed GABA signaling in ASD (Al-Otaish et al., 2018). The discovery that estrogens rescue ASD phenotypes in both zebrafish and mouse models of autism is an especially convincing piece of evidence for the female protective theory (Macrì et al., 2010; Hoffman et al., 2016).

It is also possible that the female sex is not protective, but males are particularly vulnerable. Three studies of gene expression patterns noted males generally had a higher expression of genes implicated in ASD, such as chromatin regulators and genes related to immune involvement (Ziats and Rennert, 2013; Shi et al., 2016; Werling et al., 2016). A study with rat models of ASD reported male-specific downregulation of *MeCP2* leading to abnormal glutamate activity, providing another potential mechanism for male-specific vulnerability (Kim et al., 2016). Interestingly, multiple studies have found decreased levels of aromatase – an enzyme that catalyzes the conversion of testosterone to estradiol – in the brains of adolescent ASD individuals (Sarachana et al., 2011; Crider et al., 2014). Decreased aromatase has also been associated with decreased RAR-related orphan receptor A (*RORA*), an ASD-associated gene that is oppositely regulated by male and female hormones (Nguyen et al., 2010; Sarachana et al., 2011). Hu et al. (2015) found a much stronger correlation between *RORA* expression and that of its targets in the cortex of male mice relative to female mice, suggesting that RORA-deficient males may have greater dysregulation of genes than females.

Of course, there may also be a combination of female-specific protective and male-specific deleterious effects. For example, Jung et al. (2018) recently assessed sexually dimorphic traits in a CHD8^+/N2373*K*^ mouse model of autism. While male mice demonstrated abnormal social behaviors such as isolation-induced self-grooming, female behavior was similar to controls. Neuronal excitability was also enhanced in males and suppressed in females. Transcriptomes were distinct, with female mice revealing an enrichment for ECM molecules, likely providing a protective effect.

A likely mechanism of divergent modulation is from differential effects of sex hormones, which have been hypothesized to play an important role in ASD pathology for both males and females (Baron-Cohen et al., 2005, 2015; Whitehouse et al., 2010; Honk et al., 2011; Ferri et al., 2018). For example, testosterone and estrogen have been shown to have contrasting effects on the immune system (Lenz et al., 2013; Roved et al., 2017), which has been repeatedly shown to play a pathological role in ASD (Estes and McAllister, 2015; Koyama and Ikegaya, 2015; Kim et al., 2017; McCarthy and Wright, 2017; Nadeem et al., 2019). Schwarz et al. (2011) analyzed biomarkers from individuals with Asperger’s syndrome and found 24 male-specific and 17 female-specific hits, including many immune-related molecules. Spine density, another phenotype strongly implicated in autism (Comery et al., 1997; Irwin et al., 2001; Hutsler and Zhang, 2010; Durand et al., 2012; Takuma et al., 2014; Tang et al., 2014; Liu et al., 2017a, b; Soltani et al., 2017), is also affected by testosterone (Hatanaka et al., 2015). Key molecules involved in neurotransmission such as GABA, glutamate, serotonin, and BDNF are all implicated in ASD and modulated by sex hormones (Kim et al., 2016, p. 2; Saghazadeh and Rezaei, 2017; Al-Otaish et al., 2018; Edwards et al., 2018; Ferri et al., 2018; Garbarino et al., 2018; Zieminska et al., 2018). It is not yet clear whether the majority of differences between male and female presentation of ASD arise from differential regulatory actions of sex hormones or from other modifiers, but the presence of a sexually dimorphic phenotype is well established. Future research will likely elucidate a clearer picture of the identity and mechanisms of sex-specific modifiers.

## Clinical Implications and Future Perspectives

**FIGURE 1:**
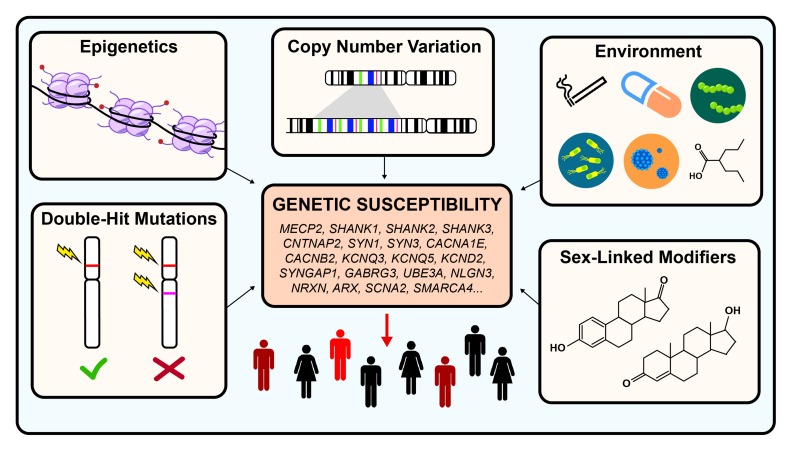
Genetic modifiers in autism spectrum disorder. Autism is estimated to be 40–80% heritable. However, both genetic and non-genetic factors modulate the penetrance of risk genes, resulting in a highly heterogeneous disease phenotype for similar pathogenic variants. Examples of genetic modulators include CNV, epigenetics, and double-hit mutations. Examples of non-genetic modifiers include environmental exposures and sex-linked modifiers.

When autism was first described, it was hypothesized to be an environmentally caused disease. Decades of research have since revealed that autism is a highly heterogeneous and extremely complex genetic condition. Even though great progress had been made in identifying hundreds of risk genes, very little is known about the different types of modifiers that may exacerbate or ameliorate disease severity. Such modifiers could include epigenetics, sex-linked modifiers, CNVs, double-hit mutations, or environmental factors (see Figure 1).

It may take many more decades of research before the scientific community has an accurate picture of how these modulators contribute to the etiology of ASD. However, this understanding is critical for the development of effective therapies. Due to the extremely diverse genetic phenotype of patients, personalized medicine may be a future avenue for maximally effective treatment. A condensed series of genetic tests – such as a microarray with identified risk loci – could be an expedient and cost-effective solution to determining genetic etiology. Alternatively, therapies may be developed to address convergent disease phenotypes that encompass multiple genetic etiologies, such as neuronal hyperexcitability and abnormal synaptic function. Autism research has come astonishingly far in just a half a century. There is much more work to be done, but continued investigation will eventually lead to a cohesive understanding of the interplay between causative genetic factors and disease modifiers in the etiology of ASD.

## Author Contributions

LR contributed to writing the main text and gathering all references. AG-G contributed to editing the manuscript drafts and providing insight into structure and material that should be included.

## Conflict of Interest Statement

The authors declare that the research was conducted in the absence of any commercial or financial relationships that could be construed as a potential conflict of interest.
